# Combined radiation and immune checkpoint inhibitor therapy for metastatic or recurrent hepatocellular carcinoma: a real-world study of 108 patients

**DOI:** 10.3389/fimmu.2025.1594577

**Published:** 2025-08-04

**Authors:** Yirui Zhai, Yefan Zhang, Fan Wu, Jianguo Zhou, Lingxia Xin, Feng Ye, Wei Sun, Huiying Zeng, Yan Song, Yongkun Sun, Wen Zhang, Shu-Lian Wang, Yuan Tang, Hui Fang, Pan Zhao, Yueping Liu, Ningning Lu, Shunan Qi, Hao Jing, Wenwen Zhang, Yongwen Song, Ye-Xiong Li, Liming Wang, Bo Chen

**Affiliations:** ^1^ Department of Radiation Oncology, National Cancer Center/National Clinical Research Center for Cancer/Cancer Hospital, Chinese Academy of Medical Sciences and Peking Union Medical College, Beijing, China; ^2^ Department of Hepatobiliary Surgery, National Cancer Center/National Clinical Research Center for Cancer/Cancer Hospital, Chinese Academy of Medical Sciences and Peking Union Medical College, Beijing, China; ^3^ Department of Radiology, National Cancer Center/National Clinical Research Center for Cancer/Cancer Hospital, Chinese Academy of Medical Sciences and Peking Union Medical College, Beijing, China; ^4^ Department of Interventional Therapy, National Cancer Center/National Clinical Research Center for Cancer/Cancer Hospital, Chinese Academy of Medical Sciences and Peking Union Medical College, Beijing, China; ^5^ Department of Medical Oncology, National Cancer Center/National Clinical Research Center for Cancer/Cancer Hospital, Chinese Academy of Medical Sciences and Peking Union Medical College, Beijing, China

**Keywords:** hepatocellular carcinoma, radiotherapy, immunotherapy, immune checkpoint inhibitor, PD-1/PD-L1 inhibitor

## Abstract

**Background:**

The prognosis of metastatic or recurrent hepatocellular carcinoma (HCC) remains poor, and new treatment strategies are warranted. Despite promising preclinical results demonstrating that radiation primes the immune system and produces a synergistic antitumor response for long-term disease control, limited clinical data are available. Therefore, in this study, we investigated the efficacy and safety of combining radiotherapy with immune checkpoint inhibitors (ICIs) for patients with metastatic and recurrent HCC in a real-world setting.

**Materials and methods:**

Patients with stage IV or recurrent HCC who received sequential or concurrent radiotherapy and ICIs in our institution were enrolled in this study. Data regarding clinicopathological characteristics, treatment protocols, response rates, toxicities, and survival were collected.

**Results:**

From January 2018 to December 2021, 108 patients were included. Extra-hepatic metastasis and portal vein tumor thrombosis were recorded in 58 (53.7%) and 69 patients (63.9%), respectively. A median radiation dose of 55 Gy was administered, and ICIs were administered for 3 weeks until disease progression or limiting toxicities occurred. After treatment, the overall response rate was 75.0%, and the median follow-up duration was 18.8 months. Median overall survival and progression-free survival were 17.0 and 12.6 months, respectively. Only one patient experienced in-field recurrence. Grade ≥3 adverse events were observed in 30.6% of patients. Dermatitis was the most common toxicity-related event and thrombocytopenia was the most common grade ≥3 adverse event, occurring overall in 17.6% of patients.

**Conclusion:**

The ICI and radiotherapy combination was effective and well-tolerated in real-world patients, achieving more favorable results compared with historical ones and providing a new multimodality therapy for patients with recurrent and metastatic HCC.

## 1 Introduction

Liver cancer is one of the leading causes of cancer-related deaths worldwide, with hepatocellular carcinoma (HCC) being the most common histological type, accounting for 75–85% of all reported cases (1). Several patients with HCC develop metastatic disease that cannot be effectively treated with current curative therapies and have poor prognosis despite using standard therapeutic regimens, such as sorafenib or lenvatinib, resulting in a median survival time (MST) of only 6.5–13.6 months (2, 3). Immune checkpoint inhibitors (ICIs), particularly programmed cell death checkpoint inhibitors (anti-programmed cell death-1 [PD1]/programmed cell death ligand 1 [PD-L1]), provide an effective treatment option for various carcinomas, including HCC (4–6). Several studies have focused on administering anti-PD1/PD-L1 regimens to patients with metastatic HCC (7–11). However, findings from several studies have generated controversy, underscoring the limited impact of anti-PD1 alone on HCC (7, 9, 10, 12). Previous studies have reported better outcomes with the addition of targeted therapy to immunotherapy; however, the response (21–32%) remains limited (10, 13, 14). Therefore, there is an urgent need to explore novel treatment combinations, particularly those involving the utilization of immune sensitizers.

Evidence has demonstrated the efficacy of radiotherapy in HCC (15–19). Studies conducted on other malignancies have observed that radiation upregulates PD-L1 expression in tumor cells. Preclinical data have also shown that radiation augments the antitumor effect of immune checkpoint inhibitors in a murine HCC model (20). Therefore, combining radiation with anti-PD1/PD-L1 may increase the response rate and improve treatment outcomes. However, despite these potentially impressive theoretical advantages, large cohort data regarding the safety and efficacy of clinical practice are lacking. To better understand the activity of this new treatment combination in unselected patients with advanced and recurrent HCC, we conducted a real-world study.

## 2 Materials and methods

### 2.1 Ethics

This retrospective study was conducted in accordance with both the Declarations of Helsinki and Istanbul and was approved by the ethics committee of the National Cancer Center (No.22/094-3295).

### 2.2 Patient selection

Patients between the ages of 18 and 75 years with stage IV (according to the American Joint Committee on Cancer Staging System, 8^th^ edition) or recurrent HCC after undergoing other treatments, with at least one measurable lesion and an Eastern Cooperative Oncology Group performance status score of 0–2, Child-Pugh A5-B7, who received radiotherapy and PD-1/PD-L1 inhibitors either sequentially (interval ≤ 8 weeks) or concurrently at our institution between 2018 and 2021 were included in this study. Those with incomplete medical records were excluded. All therapeutic regimens were defined and discussed by a multidisciplinary treatment team (MDT).

### 2.3 Radiotherapy and immune checkpoint inhibitors

Four-dimensional computed tomography (CT) and magnetic resonance (MR) simulations were performed after ≥4 hours of fasting, during which patients were placed in a supine position with thermoplastic mask immobilization. All images were fused for delineation at the Pinnacle workstation (Philips Healthcare Nederland B.V., The Netherlands).

For patients with intrahepatic carcinoma, the location of the liver, portal vein, and hepatic veins was used for CT and MR image fusion; otherwise, bone alignment was utilized. For patients who received hepatic radiation, gross tumor volume (GTV) included the primary tumor and tumor thrombosis (if present), with or without regional metastatic lymph nodes evaluated using the multi-phase contrast-enhanced CT (width 350, level 40) and MR images. If possible, adjacent multifocal lesions were included.

Clinical target volume (CTV) included GTV plus a 5-mm margin and 10–15-mm expansion from the GTV toward the direction of the blood vessels with tumor thrombosis. However, for patients who received metastasis radiation, CTV often included GTV plus 5 mm in three dimensions. The planning target volume was defined as CTV plus a margin based on the movements in 4D-CT (usually a 5-mm margin in the axial and 10–15-mm in the cranial–caudal directions). For patients who received hepatic radiation, 50–65 Gy was delivered in 20–30 fractions. Intensity-modulated radiation therapy was utilized for all patients. For patients underwent radiotherapy targeting to intrahepatic lesions, we recommended the liver constraint dose as following, mean liver minus GTV dose ≤18-20Gy or the volume of liver minus GTV spare from 5Gy≥ 300ml. Radiotherapy quality assurance and control were performed for all patients. PD-1/PD-L1 inhibitors were administered every 3 weeks until disease progression, limiting toxicity or patient rejection. For patients with HBV infection, entecavir (0.5 mg Qd) was routinely administered. For patients with activated HCV infection, the antiviral regimen typically consisted of sofosbuvir (400 mg Qd) and/or velpatasvir (100 mg Qd).

### 2.4 Follow-ups

Symptoms, physical examinations, blood pressure, blood counts, and liver function were regularly assessed once a week during the radiation and at every follow-up stage. Blood counts, liver function, renal function, thyroid function, myocardial enzyme, and cortisol levels were evaluated every two cycles of immunotherapy. Neck, chest, abdominal, and pelvic CTs; hepatic MR imaging; alpha-fetoprotein (AFP), carcinoembryonic antigen, carbohydrate antigen 19-9, and ferritin levels, and the presence of hepatitis virus (hepatitis C virus [HCV]-RNA and hepatitis B virus [HBV]-DNA, only for patients with virus hepatitis) were assessed 1 month after treatment, then every 3 months for 2 years, and thereafter every 6 months for the next 3 years. For patients with suspicious abnormal symptoms, examinations would be administered at any time.

### 2.5 Statistical analysis

Data on clinicopathological characteristics, AFP levels, treatment details, response rates, toxicities, and survival were collected. Response rates were assessed according to the Response Evaluation Criteria in Solid Tumors (RECIST) 1.1 and modified Response Evaluation Criteria in Solid Tumors (mRECIST) for HCC (21). Toxicities were graded using the common terminology criteria for adverse events, version 5.0. Survival was determined from the first day of treatment delivery employing the Kaplan–Meier method. Progression-free survival (PFS) was defined as patient death or documented disease progression. In-field relapse-free survival (IRFS) was measured as the time until relapse within the radiation field. Out-field relapse-free survival (ORFS) was measured as the time to metastasis or recurrence outside the radiation field. Univariate analyses were conducted, including variables such as sex, age, alcohol use, viral hepatitis, stage, time from the immunotherapy to the radiotherapy, and target therapy, employing the log-rank test.

## 3 Results

### 3.1 Characteristics

Between January 2018 and December 2021, 108 patients—100(92.6%) of whom were males with a median age of 56.5 years—met the inclusion criteria (**Supplementary Figure 1**). The median pretreatment AFP value in the entire group was 406 ng/mL (1.51–178376 ng/mL). Forty-three (39.8%) patients had AFP levels ≥ 1,000 ng/mL. Additionally, the median tumor maximum diameter was 8.2 cm (1.2–23.2 cm). Among the patients, 91 (84.3%) showed disease progression with previous treatment, including 19 patients (17.6%) who experienced disease progression after receiving PD1/PD-L1 inhibitors. There were 69 patients (63.9%) with portal vein tumor thrombosis, including 30 patients (27.8%) with main portal vein tumor thrombus. Extra-hepatic metastasis was recorded in 58 patients (53.7%). Patient characteristics are listed in **Table 1**.

**Table 1:** Characteristics of enrolled patients.

Characteristics	No.	%	Characteristics	No.	%
Sex			ECOG		
Male	100	92.6	1	107	99.1
Female	8	7.4	2	1	0.9
Age (years)	56.5 (31–74)		Diagnosis		
Child–Pugh			Pathological	88	81.5
5	88	81.5	Clinical	20	18.5
6	16	14.8	Hepatocirrhosis		
7	4	3.7	Yes	81	75.0
Hepatitis			No	27	25.0
HBV	88	81.5	Alcohol		
HCV	6	5.6	Yes	47	43.5
No	14	13.0	No	61	56.5
AFP^^^			No. of tumors		
≥ 1,000	43	39.8	1–4	71	65.7
< 1,000	65	60.2	≥ 5	37	34.3
EHM			PVTT		
Yes	58	53.7	Yes	69	63.9
No	50	46.3	No	39	36.1
IVTT			VP4		
Yes	12	11.1	Yes	30	27.8
No	96	88.9	No	78	72.2

AFP, alpha-fetoprotein; EHM, extra-hepatic metastasis before enrollment; ECOG, Eastern Cooperative Oncology Group performance status; HBV, hepatitis B; HCV, hepatitis C; PVTT, portal vein tumor thrombosis; VP4, tumor thrombus in main portal vein; IVTT, tumor thrombus in inferior vena cava ^^^ng/mL.

### 3.2 Treatment

Radiation and PD-1/PD-L1 inhibitors were used as first-line, second-line, and ≥ third-line therapies in 17 (15.7%), 47 (43.5%), and 44 (40.7%) patients, respectively. All patients received immunotherapy every 3 weeks. One patient experienced an allergic reaction during camrelizumab injection and was subsequently switched to toripalimab. The median duration of ICIs was 10.5 cycles (1-36 cyles). Radiation could be delivered to more than one site in each patient. In total, radiotherapy was administered to 225 foci. The median actual radiation dose, median biological equivalent dose, and equivalent dose of 2 Gy per fraction(α/β=10Gy) were 55 Gy (25–65 Gy), 71 Gy (38–100 Gy), and 60 Gy (31–83 Gy), respectively. In most patients, radiation was delivered to intrahepatic tumors, including those with or without portal vein tumor thrombosis (PVTT). Additionally, 93 patients (86.1%) received targeted therapy, whereas 94 (87.0%) received antiviral therapy during radiotherapy and immunotherapy (**Table 2**).

**Table 2:** Treatment details.

Treatment	No.	%	Treatment	No.	%
Treatment mode			RT sites^#^		
Concurrent	47	43.5	Primary tumor	81	75.0
Sequential	61	56.5	PVTT	62	57.4
Current therapy line			Other TT	10	9.3
1	17	15.7	Lymph node	21	19.4
2	47	43.5	Others metastasis	51	47.2
3	21	19.4	Targeted therapy^^^		
4	15	13.9	Sorafenib	29	26.9
5	4	3.7	Lenvatinib	48	44.4
6	4	3.7	Regorafenib	13	12.0
ICI			Apatinib	3	2.8
Toripalimab^*^	42	38.9	No	15	13.9
Sintilimab	32	29.6	RT technique		
Camrelizumab^*^	19	17.6	IMRT	4	3.7
Tislelizumab	10	9.3	VMAT	101	93.5
Pembrolizumab	3	2.8	TOMO	3	2.8
Nivolumab	1	0.9	Anti-virus		
Zimberelimab	1	0.9	Yes	94	87.0
Atezolizumab	1	0.9	No	14	13.0

ICI, immune checkpoint inhibitor; IMRT, intensity modulated radiation therapy; PVTT, portal vein tumor thrombosis; RT, radiotherapy; TOMO, tomotherapy; TT, tumor thrombosis; VMAT, volumetric modulated arc therapy.

^*^One patient experienced an allergic reaction during the camrelizumab injection and was subsequently switched to toripalimab.

^#^Radiation could be delivered to more than one site in each patient.

^^^Concurrent with an immune checkpoint inhibitor.

### 3.3 Outcomes

**Figure 1:**
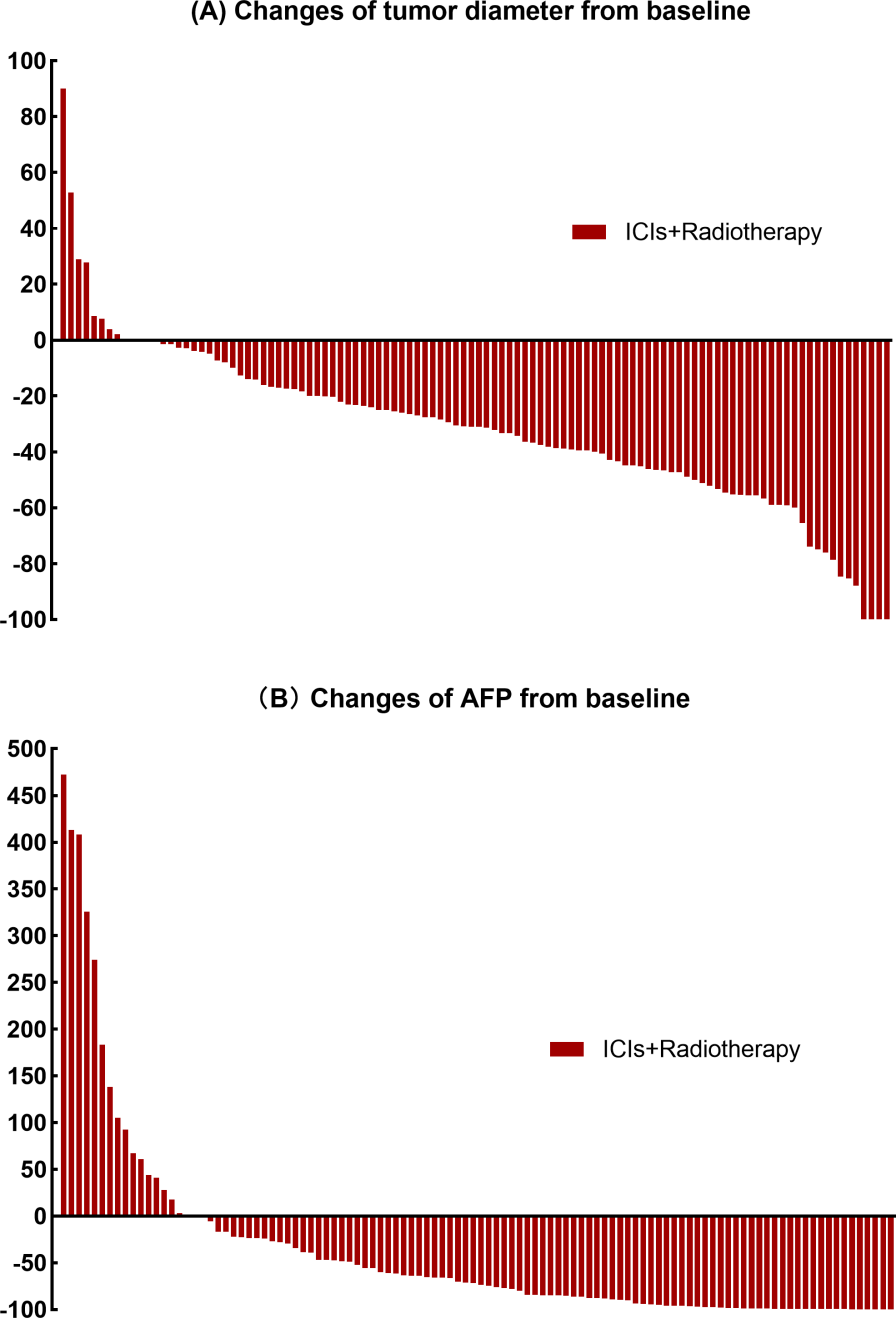
**(A)** Percentage change from baseline in the sum of the diameters of the target lesions. **(B)** Percentage change from baseline levels of alpha-fetoprotein.

The findings regarding objective response rate (ORR), complete response (CR), and partial response (PR) according to RECIST 1.1. and mRECIST are outlined in **Table 3** and **Figure 1A**. AFP decrement was observed in 81.5% of patients, and the decreasing ratio is depicted in **Figure 1B**. The median AFP levels after treatment were measured at 44.02 ng/mL. Moreover, 85 patients (78.7%) had AFP levels ≤400 ng/mL after treatment. No statistically significant correlation between AFP reduction and objective response rate (RECIST 1.1: CR+PR p=0.088; mRECIST: CR+PR p=0.337) or disease control rate (RECIST 1.1: CR+PR+SD p=0.087; mRECIST: CR+PR+SD p=0.263) was revealed.

**Table 3:** Response to radiation and immunotherapy.

Target	All					In-field tumor only				
Criteria	RECIST		mRECIST		p	RECIST		mRECIST		p
	No.	%	No.	%		No.	%	No.	%	
CR	4	3.7	19	17.6	–	14	13.0	27	25.0	–
PR	53	49.1	62	57.4	–	84	77.8	73	67.6	–
SD	48	44.4	25	23.1	–	10	9.3	8	7.4	–
PD	3	2.8	2	1.9	–	0	0.0	0	0.0	–
ORR	57	52.8	81	75.0	<0.001	98	90.7	100	92.6	<0.001
DCR	105	97.2	106	98.1	<0.001	108	100.0	108	100.0	1.000

CR, complete response; DCR, disease control rate; mRECIST, modified response evaluation criteria in solid tumor; ORR, objective response rate; PD, progressive disease; PR, partial response; RECIST, response evaluation criteria in solid tumor; SD, stable disease

**Figure 2:**
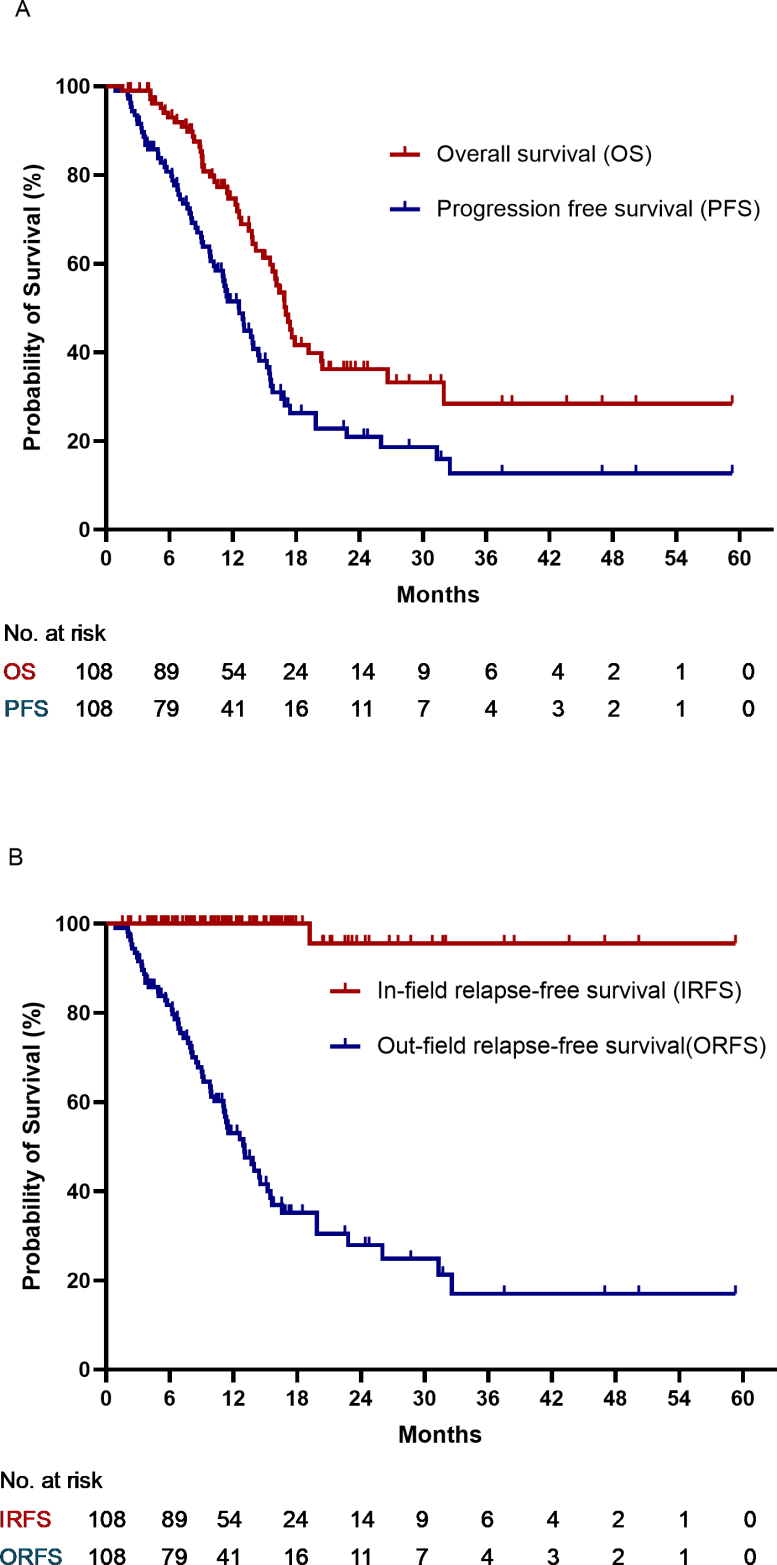
**(A)** Overall survival and progression-free survival. **(B)** In-field relapse-free survival and out-field relapse-free survival.

The median follow-up time was 18.8 months (4.3–62.6 months). At the last follow-up visit, 49 patients had died: 40 due to tumors, two due to adverse events (AEs) (one related to immunotherapy), and seven due to other diseases. Progressive disease was recorded in 63 patients, including 62 with out-of-radiation field progression and one with both in-field and out-of-field relapses. The median values for overall survival (OS), PFS, and ORFS were 17.0 (95%CI 15.6-18.0), 12.6(95%CI 10.8-14.5), and 13.3 (95%CI 10.5-15.5) months, respectively. The median IRFS was not achieved. The 1- and 2-year rates were: OS, 74.7% and 36.2%; PFS, 51.5% and 20.9%; IRFS, 100% and 96.7%; and ORFS, 51.7% and 27.9%, respectively. The survival curves are plotted in **Figures 2A, B**.

### 3.4 AEs

Dermatitis was the most common form of toxicity, followed by leukopenia. Hypothyroidism, a typical immune-related AE, was observed in 29 patients (26.9%). Notably, no viral reactivation (HBV/HCV) was observed during the study. Grade≥2 pneumonitis was observed in 4 patients (3.7%). There is no radiation induced liver injury or immune-related liver injury. One patient died of a tracheoesophageal fistula induced by immunotherapy and target therapy, while another of severe diarrhea induced by lenvatinib. The remaining patients with AEs were cured after the treatment (**Table 4**).

**Table 4:** Adverse events from any causes.

AEs	Grade 1		Grade 2		Grade 3		Grade 4		Grade 5	
	No.	%	No.	%	No.	%	No.	%	No.	%
Dermatitis	91	84.3	4	3.7	3	2.8	0	0.0	0	0.0
GI AE	22	20.4	16	14.8	6	5.6	0	0.0	1	0.9
Pneumonitis	1	0.9	1	0.9	3	2.8	0	0.0	0	0.0
Hypertension	0	0.0	1	0.9	0	0.0	0	0.0	0	0.0
Abnormal COR	0	0.0	0	0.0	1	0.9	0	0.0	0	0.0
Arrhythmia	0	0.0	1	0.9	1	0.9	0	0.0	0	0.0
Hypothyroidism	23	21.3	3	2.8	2	1.9	1	0.9	0	0.0
TEF	0	0.0	0	0.0	0	0.0	0	0.0	1	0.9
Abnormal ALT	20	18.5	12	11.1	0	0.0	0	0.0	0	0.0
Abnormal AST	28	25.9	16	14.8	2	1.9	0	0.0	0	0.0
Renal Injury	2	1.9	2	1.9	0	0.0	0	0.0	0	0.0
Leucopenia	42	38.9	31	28.7	6	5.6	1	0.9	0	0.0
Anemia	30	27.8	4	3.7	0.0	0	0.0	0	0	0.0
TBC	27	25.0	22	20.4	16	14.8	3	2.8	0	0.0

ALT, alanine transaminase; AST, aspartate aminotransferase; COR, cortisol; GI AE, gastrointestinal adverse events; TBC, thrombocytopenia; TEF, tracheoesophageal fistula.

### 3.5 Prognosticators

Patients who previously received ICIs had a worse OS than those who did not. Still, patients who received ICIs before achieved promising MST (11.4 months) and mPFS (9.0 months). Factors such as sex, age, alcohol use, hepatitis, hepatocirrhosis, AFP levels, PVTT, number of tumors, distant metastases, time from the immunotherapy to the radiotherapy, radiation dose and radiotherapy fractionation were not associated with PFS (**Table 5**). Outcomes were consistent across different ICIs regimens (toripalimab vs. sintilimab vs. others, PFS, p=0.179; OS, p=0.448).

**Table 5:** Prognostic factors in univariate analysis.

Prognostic factor	MST (months)			mPFS (months)		
	Yes	No	p	Yes	No	p
Male	17.2	10.5	0.205	12.9	7.7	0.300
Age<60 years	16.9	17.0	1.000	11.4	13.0	0.461
Alcohol use	17.9	16.4	0.282	11.0	13.0	0.559
Hepatitis	17.0	16.9	0.851	13.0	12.6	0.701
Hepatocirrhosis	17.4	16.4	0.789	12.9	11.4	0.707
AFP <1000	17.4	16.9	0.303	12.6	11.4	0.524
No. of tumors ≥5	15.5	17.2	0.111	9.9	13.7	0.113
EHM	17.6	16.1	0.567	12.9	11.3	0.587
PVTT	16.1	20.5	0.102	11.2	13.7	0.116
VP4	14.9	17.0	0.312	9.2	13.7	0.150
ICI before	11.4	17.4	0.024	9.0	13.0	0.086
Concurrent	14.9	17.6	0.114	9.9	14.0	0.059
RIHL	17.0	16.0	0.845	12.9	11.5	0.343
SBRT	16.0	17.0	0.732	10.4	13.7	0.153
EQD2<60Gy	17.9	16.4	0.959	11.5	12.6	0.322
Target therapy	16.9	NR	0.143	12.6	9.9	0.974

AFP, alpha-fetoprotein; EHM, extrahepatic metastasis before enrollment; EQD2, equivalent dose for 2Gy per fraction(α/β=10Gy), ICI, immune checkpoint inhibitor; mORFS, median out-radiation field relapse-free survival; mPFS, median progression-free survival time; MST, median survival time; NR, not reached; PVTT, portal vein tumor thrombosis; RIH, radiotherapy for intrahepatic lesions; SBRT, stereotactic body radiation therapy; VP4, tumor thrombus in the main portal vein.

## 4 Discussion

Our study showed a promising response rate, favorable survival rate, rare radiation field relapse, and tolerable AEs. Additionally, this study indicated that combined radiation and anti-PD1 was an efficient and tolerable treatment for these patients.

Our results were compared with those of previous studies (**Supplementary Table 1**). Notably, previous studies have utilized other treatments as first- or second-line therapies, whereas in our study, more than 40% of patients received the current therapy as third-line or subsequent treatments. In comparison to classical multi-kinase inhibitors, such as sorafenib (9.2–11%),lenvatinib (24.1%) and regorafenib (2, 7, 14, 22) (23), our study demonstrated a significantly higher response rate(52.8% according to RECIST, 75.0% according to mRECIST). Moreover, when compared with anti-PD1 therapies alone (7, 10, 14, 22, 24, 25) (9), our study exhibited a clear advantage. Additionally, we achieved the highest disease control rate (97.5% according to RECIST, 98.1% according to mRECIST) compared with previous studies (55–75.5%) (7, 10, 14, 22, 24, 25). Durable responses and prolonged survival times were observed in this study. Compared with single multi-kinase inhibitors, anti-angiogenic therapy, and immunotherapy options—which were also employed during subsequent-line therapy—our results demonstrated a significantly extended median PFS (mPFS) (12.6 months vs. 2.1–4.9 months) (2, 8, 23, 25–28). The mPFS achieved in our study was better than first-line systemic therapies using sorafenib or lenvatinib (4.3–7.4 months) and r (2, 14). Moreover, our 1-year PFS rate (51.5%) surpassed that of the KEYNOTE 220 and KEYNOTE-224 trial using pembrolizumab (28%) and the CheckMate-459 trial using nivolumab (22%) (7, 28). The MST (17.0 months) in our study outperformed those of previous studies with multi-kinase inhibitors (8.5–14.7 months), cabozantinib (10.2 months), pembrolizumab (12.9–13.9 months), durvalumab (13.8 months), and camrelizumab (15.1 months) (9, 24, 25, 27). These comparisons demonstrate that the PFS post-immunotherapy alone is limited and that the addition of radiation can enhance the antitumor effect. Our study included some patients with progressive disease after immunotherapy, and we found that the addition of radiotherapy might reverse immunotherapy resistance subsequently achieving profitable PFS.

Compared with treatments of anti-PD-1/PD-L1 regimens combined with various of other systemic therapies involving cytotoxic T lymphocyte-associated antigen-4 antibody, multi-kinase or anti-angiogenesis agents, anti-PD-1/PD-L1 therapies plus radiation have demonstrated a higher tumor response rate (52.8% vs. 24–32% according to RECIST, 75.0% vs. 30.0–46.0% according to mRECIST), better disease control (98.1% vs. 75% according to mRECIST), and longer PFS (12.6 months vs. 2.2–6.9 months) (7, 8, 10, 14, 22, 25) (29). However, this superiority does not extend to MST (17.0 months vs. 12.0–22.8 months). Two factors account for this difference: First, there is an imbalance in the treatment history between the patients in our study and those in other studies. As previously mentioned, more than 80% of patients in our study had already received multiple lines of therapy, with 17% having undergone anti-PD-1/PD-L1 therapy. Consequently, limited salvage therapeutic regimens were available after disease progression. Second, our study enrolled more patients with adverse prognostic factors, such as PVTT (63.9%), main trunk of portal vein tumor thrombosis (Vp4) (27.8%), hepatocirrhosis (75.0%), viral hepatitis (87.1%), and higher AFP levels: 39.8% of patients with AFP ≥ 1,000 ng/mL, 50% of patients with AFP ≥ 400 ng/mL in our study, and 38% of patients with AFP ≥ 400 ng/mL in the IMbrave150 study (14).

Previous studies have consistently demonstrated that PVTT is a negative prognostic factor for HCC (30). However, we observed that some of the other studies listed PVTT, especially Vp4, as an exclusion criterion (29), whereas our study enrolled more than 60% of patients with PVTT and nearly one-third with Vp4. Nevertheless, our combined therapy approach showed more promising survival outcomes compared with historical studies, even with the disadvantage of these baseline characteristics (2, 8, 9, 28). Importantly, we found no significant difference in survival between patients with and without PVTT. The median survival of patients with PVTT was superior to that reported in previous studies utilizing targeted therapies, immunotherapies, or other combinations (31). Even patients with Vp4 disease showed favorable MST (14.9 months) and mPFS (9.2 months), compared with the Vp4 subgroup analysis in the IMbrave150 study (MST 7.6 months, mPFS 5.4 months), which used atezolizumab and bevacizumab (32). Two factors might have contributed to the phenomenon. First, previous studies have demonstrated that PVTT has an unsatisfactory prognosis due to the increasing difficulty of performing operations and interventional therapy, without considering radiation. Second, patients with PVTT might be sensitive to radiotherapy due to the abundant oxygen supply from diffusive blood circulation at this site.

Several previous studies on PD-1/PD-L1 antibodies have excluded patients with viral hepatitis due to the potential for virus reactivation during treatment. However, viral hepatitis accounts for > 60% of HCC cases in China, and excluding it from the study would reduce its representativeness (33). Meanwhile, virus reactivation after anti-PD1 therapies is approximately 2%–5% (34, 35), which is relatively acceptable according to historical reports. In a phase III trial, KEYNOTE-394, 78.7% of patients with HBV infection were enrolled, and immunotherapy-induced hepatitis occurred in only 1.7% of the patients (36). Therefore, in real-world practice, we did not prohibit ICI delivery to patients with hepatitis. Theoretically, patients with hepatitis might benefit more from PD1/PD-L1 antibodies based on the phenomenon that high levels of T cells in HBV-infected patients may enhance the sensitivities of PD-1 antibodies, and PD-1 antibodies could also evoke the immune system to defend against the virus (37). In contrast, other studies have shown that the virus-related HCC microenvironment is more immunosuppressive and exhausting than the non-viral microenvironment (38). However, these two theories have not been proven in clinical practice. We found that the survival rates in patients with hepatitis were equivalent to those of other patients, consistent with the results of other studies (10, 13, 28, 36). Overall, our study safely administered immunotherapy to patients with HBV/HCV infection with the protection of antiviral therapies.

Grade ≥ 3 AEs occurred in 30.6% of patients, consistent with the findings of a study that prescribed anti-PD-1/PD-L1 regimens alone (22–68%) (7–10, 25, 28). Most AEs involved hematological toxicities that resolved with treatment. Two patients experienced Grade 5 AEs, and one developed severe diarrhea following lenvatinib, and the patient declined any further intervention. That patient received abdominal radiation targeting to intrahepatic lesion and PVTT with dose of 27.5Gy in 5 fractions. Maximum point doses to the intestine (18.6 Gy) and colon (23.8 Gy) over 5 fractions were substantially below tolerance thresholds (35 Gy and 33 Gy respectively). Given that diarrhea is a common treatment-emergent AE among lenvatinib-treated patients, lenvatinib may be the direct cause. The other patient was diagnosed with rib and vertebral metastases after transarterial chemoembolization and sorafenib treatment; subsequently, the patient received concurrent toripalimab, regorafenib, and radiation to bone metastasis at a dose of 40 Gy in 10 fractions. A tracheoesophageal fistula was observed 11 months after radiation therapy and during maintenance therapy with toripalimab and regorafenib. Considering the very reasonable esophageal dose (maximum dose to the esophagus: 33 Gy and V32 < 10 mL with a conventional fraction) and the extended interval between radiation and the AE, radiation was less responsible for the fistula. On the contrary, previous studies reported gastrointestinal perforation in PD-1 inhibitor and regorafenib (39, 40). We inferred that toripalimab or regorafenib might have induced the observed toxicity. Recently, RTOG 1112 demonstrated adding SBRT improved OS (15.8 months) and PFS (9.2 months) in patients with advanced HCC, compared to sorafenib alone without the increment of AEs. Another prospective study proposed by our team also showed satisfied OS (16.5 months) and PFS (6.1 months) with tolerable toxicities after receiving concurrent radiotherapy and sorafenib in advanced HCC patients (41, 42). Furthermore, the combination of radiotherapy and immunotherapy represents an actively investigated synergistic approach in oncology. Radiotherapy has been demonstrated to elicit systemic antitumor immunity, inducing responses in both irradiated lesions and non-irradiated sites – the abscopal effect phenomenon – which appears potentiated by anti-PD-1 therapy (43). Therefore, it is reasonable to infer that combined radiation with either immunotherapy or targeted therapy is safe, while a combination of tri-treatment, including immunotherapy, radiation, and targeted therapy should be administered with caution and tailored to individual patients.

The mechanism of radiotherapy induces an abscopal tumor-specific immune response in both the irradiated and nonirradiated tumors, which is potentiated by PD-1 blockade and the

The strength of our study is multifold. First, our study demonstrated favorable response rates and improved survival despite enrolling patients with negative prognosticators who had already progressed after multiple lines of therapy. Second, most patients achieved satisfactory control of the radiation field and exhibited reliable radiation effects. Third, the combined treatment decision was discussed and made by an MDT, underscoring the advantages of multimodality therapy.

However, our study has some limitations. First, as a retrospective study, we could not obtain data on patients’ PD1 and PD-L1 expressions. The predictive value of tumor cell PD1/PD-L1 expression in terms of response and prognosis remains controversial, as evidenced by contrasting results from the CheckMate 040 and CheckMate 459 studies. Second, our study employed multiple anti-PD1/PD-L1 drugs, which makes it challenging to determine the specific effects of individual drugs. Future studies should use more consistent drug types to reduce variation. Furthermore, a direct comparison to studies involving different therapeutic approaches may introduce inevitable bias due to variations in patient enrollment criteria.

### 4.1 Conclusion

Combining immune checkpoint inhibitors and radiotherapy for advanced and relapsed HCC resulted in prolonged PFS and OS with tolerable AEs, suggesting it is efficient and safe. Consequently, we proposed a randomized controlled trial comparing radiotherapy plus immunotherapy to sorafenib in advanced HCC (NCT04709380). Future studies should consider the optimal timing for treatment administration and focus on the analysis of potentially predictive biomarkers, such as PD-1/PD-L1. Furthermore, considering the promising outcomes of the IMbrave150 trial, which utilized atezolizumab plus bevacizumab, exploring the combination of radiation and the two regimens is necessary.

## Data Availability

The raw data supporting the conclusions of this article will be made available by the authors, without undue reservation.

## References

[1] Siegel RL Miller KD Fuchs HE Jemal A. Cancer statistics, 2022. CA Cancer J Clin. (2022) 72:7–33. doi: 10.3322/caac.21708, PMID: 35020204

[2] Kudo M Finn RS Qin S Han KH Ikeda K Piscaglia F. Lenvatinib versus sorafenib in first-line treatment of patients with unresectable hepatocellular carcinoma: a randomised phase 3 non-inferiority trial. Lancet. (2018) 391:1163–73. doi: 10.1016/S0140-6736(18)30207-1, PMID: 29433850

[3] Cheng AL Kang YK Chen Z Tsao CJ Qin S Kim JS. Efficacy and safety of sorafenib in patients in the Asia-Pacific region with advanced hepatocellular carcinoma: a phase III randomised, double-blind, placebo-controlled trial. Lancet Oncol. (2009) 10:25–34. doi: 10.1016/S1470-2045(08)70285-7, PMID: 19095497

[4] Faivre-Finn C Vicente D Kurata T Planchard D Paz-Ares L Vansteenkiste JF. Four-year survival with durvalumab after chemoradiotherapy in stage III NSCLC-an update from the PACIFIC trial. J Thorac Oncol. (2021) 16:860–7. doi: 10.1016/j.jtho.2020.12.015, PMID: 33476803

[5] Liu SV Reck M Mansfield AS Mok T Scherpereel A Reinmuth N. Updated overall survival and PD-L1 subgroup analysis of patients with extensive-stage small-cell lung cancer treated with atezolizumab, carboplatin, and etoposide (IMpower133). J Clin Oncol. (2021) 39:619–30. doi: 10.1200/JCO.20.01055, PMID: 33439693 PMC8078320

[6] Sznol M Ferrucci PF Hogg D Atkins MB Wolter P Guidoboni M. Pooled analysis safety profile of nivolumab and ipilimumab combination therapy in patients with advanced melanoma. J Clin Oncol. (2017) 35:3815–22. doi: 10.1200/JCO.2016.72.1167, PMID: 28915085

[7] Yau T Park JW Finn RS Cheng AL Mathurin P Edeline J. Nivolumab versus sorafenib in advanced hepatocellular carcinoma (CheckMate 459): a randomised, multicentre, open-label, phase 3 trial. Lancet Oncol. (2022) 23:77–90. doi: 10.1016/S1470-2045(21)00604-5, PMID: 34914889

[8] Finn RS Ikeda M Zhu AX Sung MW Baron AD Kudo M. Phase ib study of lenvatinib plus pembrolizumab in patients with unresectable hepatocellular carcinoma. J Clin Oncol. (2020) 38:2960–70. doi: 10.1200/JCO.20.00808, PMID: 32716739 PMC7479760

[9] Finn RS Ryoo BY Merle P Kudo M Bouattour M Lim HY. Pembrolizumab as second-line therapy in patients with advanced hepatocellular carcinoma in KEYNOTE-240: A randomized, double-blind, phase III trial. J Clin Oncol. (2020) 38:193–202. doi: 10.1200/JCO.19.01307, PMID: 31790344

[10] Yau T Kang YK Kim TY El-Khoueiry AB Santoro A Sangro B. Efficacy and safety of nivolumab plus ipilimumab in patients with advanced hepatocellular carcinoma previously treated with sorafenib: the checkMate 040 randomized clinical trial. JAMA Oncol. (2020) 6:e204564. doi: 10.1001/jamaoncol.2020.4564, PMID: 33001135 PMC7530824

[11] Ziogas IA Evangeliou AP Giannis D Hayat MH Mylonas KS Tohme S. The role of immunotherapy in hepatocellular carcinoma: A systematic review and pooled analysis of 2,402 patients. Oncologist. (2021) 26:e1036–49. doi: 10.1002/onco.13638, PMID: 33314549 PMC8176986

[12] Mahn R Vogt A Kupczyk P Sadeghlar F van Beekum K Huneburg R. Programmed cell death protein 1 (PD-1)-inhibition in hepatocellular carcinoma (HCC): a single center experience. Scand J Gastroenterol. (2020) 55:1057–62. doi: 10.1080/00365521.2020.1794539, PMID: 32692941

[13] Ren Z Xu J Bai Y Xu A Cang S Du C. Sintilimab plus a bevacizumab biosimilar (IBI305) versus sorafenib in unresectable hepatocellular carcinoma (ORIENT-32): a randomised, open-label, phase 2-3 study. Lancet Oncol. (2021) 22:977–90. doi: 10.1016/S1470-2045(21)00252-7, PMID: 34143971

[14] Cheng AL Qin S Ikeda M Galle PR Ducreux M Kim TY. Updated efficacy and safety data from IMbrave150: Atezolizumab plus bevacizumab vs. sorafenib for unresectable hepatocellular carcinoma. J Hepatol. (2022) 76:862–73. doi: 10.1016/j.jhep.2021.11.030, PMID: 34902530

[15] Chen B Wu JX Cheng SH Wang LM Rong WQ Wu F. Phase 2 study of adjuvant radiotherapy following narrow-margin hepatectomy in patients with HCC. Hepatology. (2021) 74:2595–604. doi: 10.1002/hep.31993, PMID: 34097307 PMC8672362

[16] Chen LC Lin HY Hung SK Chiou WY Lee MS. Role of modern radiotherapy in managing patients with hepatocellular carcinoma. World J Gastroenterol. (2021) 27:2434–57. doi: 10.3748/wjg.v27.i20.2434, PMID: 34092968 PMC8160620

[17] Wang L Qiu L Ke Q Ji H Wu J. Systematic review of adjuvant external beam radiotherapy for hepatocellular carcinoma following radical hepatectomy. Radiother Oncol. (2022) 175:101–11. doi: 10.1016/j.radonc.2022.08.019, PMID: 35998838

[18] Long L Chen B Wang H Zhao Y Wu F Wang L. Survival benefit of radiotherapy following narrow-margin hepatectomy in patients with hepatocellular carcinoma: A propensity score-matched analysis based on phase II study. Radiother Oncol. (2023) 180:109462. doi: 10.1016/j.radonc.2022.109462, PMID: 36634853

[19] Munoz-Schuffenegger P Barry A Atenafu EG Kim J Brierley J Ringash J. Stereotactic body radiation therapy for hepatocellular carcinoma with Macrovascular invasion. Radiother Oncol. (2021) 156:120–6. doi: 10.1016/j.radonc.2020.11.033, PMID: 33285195

[20] Kim KJ Kim JH Lee SJ Lee EJ Shin EC Seong J. Radiation improves antitumor effect of immune checkpoint inhibitor in murine hepatocellular carcinoma model. Oncotarget. (2017) 8:41242–55. doi: 10.18632/oncotarget.17168, PMID: 28465485 PMC5522235

[21] Lencioni R Llovet JM. Modified RECIST (mRECIST) assessment for hepatocellular carcinoma. Semin Liver Dis. (2010) 30:52–60. doi: 10.1055/s-0030-1247132, PMID: 20175033 PMC12268942

[22] Galle PR Finn RS Qin S Ikeda M Zhu AX Kim TY. Patient-reported outcomes with atezolizumab plus bevacizumab versus sorafenib in patients with unresectable hepatocellular carcinoma (IMbrave150): an open-label, randomised, phase 3 trial. Lancet Oncol. (2021) 22:991–1001. doi: 10.1016/S1470-2045(21)00151-0, PMID: 34051880

[23] Bruix J Qin S Merle P Granito A Huang YH Bodoky G. Regorafenib for patients with hepatocellular carcinoma who progressed on sorafenib treatment (RESORCE): a randomised, double-blind, placebo-controlled, phase 3 trial. Lancet. (2017) 389:56–66. doi: 10.1016/S0140-6736(16)32453-9, PMID: 27932229

[24] Qin S Ren Z Meng Z Chen Z Chai X Xiong J. Camrelizumab in patients with previously treated advanced hepatocellular carcinoma: a multicentre, open-label, parallel-group, randomised, phase 2 trial. Lancet Oncol. (2020) 21:571–80. doi: 10.1016/S1470-2045(20)30011-5, PMID: 32112738

[25] Kelley RK Sangro B Harris W Ikeda M Okusaka T Kang YK. Safety, efficacy, and pharmacodynamics of tremelimumab plus durvalumab for patients with unresectable hepatocellular carcinoma: randomized expansion of a phase I/II study. J Clin Oncol. (2021) 39:2991–3001. doi: 10.1200/JCO.20.03555, PMID: 34292792 PMC8445563

[26] Zhu AX Kang YK Yen CJ Finn RS Galle PR Llovet JM. Ramucirumab after sorafenib in patients with advanced hepatocellular carcinoma and increased alpha-fetoprotein concentrations (REACH-2): a randomised, double-blind, placebo-controlled, phase 3 trial. Lancet Oncol. (2019) 20:282–96. doi: 10.1016/S1470-2045(18)30937-9, PMID: 30665869

[27] Abou-Alfa GK Meyer T Cheng AL El-Khoueiry AB Rimassa L Ryoo BY. Cabozantinib in patients with advanced and progressing hepatocellular carcinoma. N Engl J Med. (2018) 379:54–63. doi: 10.1056/NEJMoa1717002, PMID: 29972759 PMC7523244

[28] Zhu AX Finn RS Edeline J Cattan S Ogasawara S Palmer D. Pembrolizumab in patients with advanced hepatocellular carcinoma previously treated with sorafenib (KEYNOTE-224): a non-randomised, open-label phase 2 trial. Lancet Oncol. (2018) 19:940–52. doi: 10.1016/S1470-2045(18)30351-6, PMID: 29875066

[29] Llovet JM Kudo M Merle P Meyer T Qin S Ikeda M. Lenvatinib plus pembrolizumab versus lenvatinib plus placebo for advanced hepatocellular carcinoma (LEAP-002): a randomised, double-blind, phase 3 trial. Lancet Oncol. (2023) 24:1399–410. doi: 10.1016/S1470-2045(23)00469-2, PMID: 38039993

[30] Jiang JF Lao YC Yuan BH Yin J Liu X Chen L. Treatment of hepatocellular carcinoma with portal vein tumor thrombus: advances and challenges. Oncotarget. (2017) 8:33911–21. doi: 10.18632/oncotarget.15411, PMID: 28430610 PMC5464922

[31] Wang K Guo WX Chen MS Mao YL Sun BC Shi J. Multimodality treatment for hepatocellular carcinoma with portal vein tumor thrombus: A large-scale, multicenter, propensity mathching score analysis. Med (Baltimore). (2016) 95:e3015. doi: 10.1097/MD.0000000000003015, PMID: 26986115 PMC4839896

[32] Breder VV Vogel A Merle P Finn RS Galle PR Zhu AX. IMbrave150: Exploratory efficacy and safety results of hepatocellular carcinoma (HCC) patients (pts) with main trunk and/or contralateral portal vein invasion (Vp4) treated with atezolizumab (atezo) + bevacizumab (bev) versus sorafenib (sor) in a global Ph III study. ASCO GI. (2021), 4073. doi: 10.1200/JCO.2021.39.15_suppl.4073

[33] Fan JH Wang JB Jiang Y Xiang W Liang H Wei WQ. Attributable causes of liver cancer mortality and incidence in China. Asian Pac J Cancer Prev. (2013) 14:7251–6. doi: 10.7314/apjcp.2013.14.12.7251, PMID: 24460283

[34] He MK Peng C Zhao Y Liang RB Lai ZC Kan A. Comparison of HBV reactivation between patients with high HBV-DNA and low HBV-DNA loads undergoing PD-1 inhibitor and concurrent antiviral prophylaxis. Cancer Immunol Immunother. (2021) 70:3207–16. doi: 10.1007/s00262-021-02911-w, PMID: 33813646 PMC10992748

[35] He MK Liang RB Zhao Y Xu YJ Chen HW Zhou YM. Lenvatinib, toripalimab, plus hepatic arterial infusion chemotherapy versus lenvatinib alone for advanced hepatocellular carcinoma. Ther Adv Med Oncol. (2021) 13:17588359211002720. doi: 10.1177/17588359211002720, PMID: 33854567 PMC8010824

[36] Qin S Chen Z Fang W Ren Z Xu R Ryoo BY. Pembrolizumab versus placebo as second-line therapy in patients from asia with advanced hepatocellular carcinoma: A randomized, double-blind, phase III trial. J Clin Oncol. (2023) 41:1434–43. doi: 10.1200/JCO.22.00620, PMID: 36455168 PMC9995104

[37] Shi F Shi M Zeng Z Qi RZ Liu ZW Zhang JY. PD-1 and PD-L1 upregulation promotes CD8(+) T-cell apoptosis and postoperative recurrence in hepatocellular carcinoma patients. Int J Cancer. (2011) 128:887–96. doi: 10.1002/ijc.25397, PMID: 20473887

[38] Lim CJ Lee YH Pan L Lai L Chua C Wasser M. Multidimensional analyses reveal distinct immune microenvironment in hepatitis B virus-related hepatocellular carcinoma. Gut. (2019) 68:916–27. doi: 10.1136/gutjnl-2018-316510, PMID: 29970455

[39] Sun C Wang X Xu Y Shao G Chen X Liu Y. Efficiency and safety of neoadjuvant PD-1 inhibitor (sintilimab) combined with chemotherapy in potentially resectable stage IIIA/IIIB non-small cell lung cancer: Neo-Pre-IC, a single-arm phase 2 trial. EClinicalMedicine. (2024) 68:102422. doi: 10.1016/j.eclinm.2024.102422, PMID: 38304743 PMC10831803

[40] Doi A Kuboki Y Shitara K Fukuoka S Bando H Okamoto W. Gastrointestinal perforation and fistula formation in 5 patients with colorectal cancer during treatment with regorafenib. Clin Colorectal Cancer. (2016) S1533-0028(16)30257-2. doi: 10.1016/j.clcc.2016.11.003, PMID: 28089507

[41] Dawson LA Winter KA Knox JJ Zhu AX Krishnan S Guha C. Stereotactic body radiotherapy vs sorafenib alone in hepatocellular carcinoma: the NRG oncology/RTOG 1112 phase 3 randomized clinical trial. JAMA Oncol. (2025) 11:136–44. doi: 10.1001/jamaoncol.2024.5403, PMID: 39699905 PMC11843352

[42] Zhai Y Wang L Zhao H Wu F Xin L Ye F. Phase II study with sorafenib plus radiotherapy for advanced HCC with portal and/or hepatic vein tumor thrombosis. JHEP Rep. (2025) 7:101287. doi: 10.1016/j.jhepr.2024.101287, PMID: 39980754 PMC11840495

[43] Ngwa W Irabor OC Schoenfeld JD Hesser J Demaria S Formenti SC. Using immunotherapy to boost the abscopal effect. Nat Rev Cancer. (2018) 18:313–22. doi: 10.1038/nrc.2018.6, PMID: 29449659 PMC5912991

