# Supplementary figures and images for: Combined radiation and immune checkpoint inhibitor therapy for metastatic or recurrent hepatocellular carcinoma: a real-world study of 108 patients
Source: Front Immunol. 2025 Aug 4;16:1594577. doi: 10.3389/fimmu.2025.1594577 (PMC12358389; doi:10.3389/fimmu.2025.1594577)

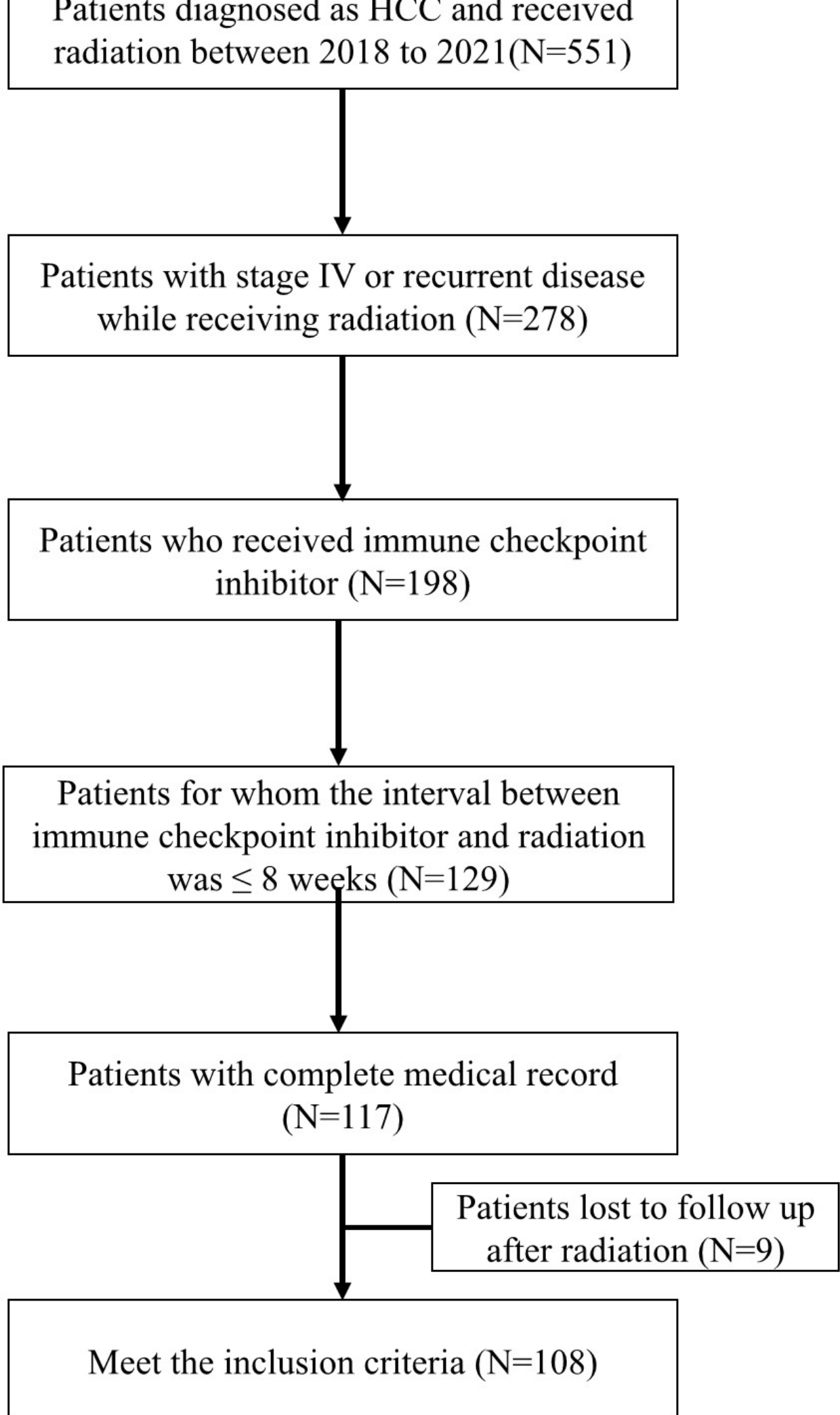

Supplement: Supplementary file 1 [file DataSheet1.pdf]
