# Supplementary material for: Combined radiation and immune checkpoint inhibitor therapy for metastatic or recurrent hepatocellular carcinoma: a real-world study of 108 patients
Source: Front Immunol. 2025 Aug 4;16:1594577. doi: 10.3389/fimmu.2025.1594577 (PMC12358389; doi:10.3389/fimmu.2025.1594577)
Supplement: Supplementary file 2 [file DataSheet2.pdf]

Supplementary Information

Combined Radiation and Immune Checkpoint Inhibitor Therapy for Metastatic or Recurrent Hepatocellular Carcinoma: A Real-World Study of 108 Patients

Short running title: Radiation and PD1 for HCC

Supplementary table. Comparative toxicity, response, and survival rates across historical trials.

Trial	Treatment	ORR (%)	DCR (%)	MST (m)	MPFS (m)	G≥3 AE %	G5 %
Current study	Radiation+ ICIs	52.8<br>75*	97.2<br>98.1*	17.0	12.6	30.6	1.9
IMbrave150[14,22]	Sorafenib	11*	55*	13.4	4.3	46	<1
Kudo et al.[2]	Sorafenib	9.2*	60.5*	12.3	3.7	67	-
Checkmate 459[7]	Sorafenib	7	58	14·7	3.9	49	<1
Kudo et al.[2]	Lenvatinib	24.1*	75.5*	13.6	7.4	75	-
RESORCE[23]	Regorafenib	11*	-	10.6	3.1	67	-
Abou-Alfa et al.[27]	Cabozantinib	4	64	10.2	5.2	68	1.3
REACH-2[26]	Ramucirumab	5	-	8.5	2.8	-	-
KEYNOTE 240[9]	Pembrolizumab	18.3	62.2	13.9	3.0	52.7	-
KEYNOTE 224[28]	Pembrolizumab	17	61	12.9	4.9	26	1
KEYNOTE 394[36]	Pembrolizumab	12.9	52.7	14.6	2.6	52.5	-
CheckMate 459[7]	Nivolumab	16	55	16·4	3.8	22	<1
Qin et al.[24]	Camrelizumab	14.7	-	13.8	2.1	22	<1
Kelley et al.[25]	Durvalumab	7.2	-	15.1	2.7	43.5	-
Kelley et al.[25]	Tremelimumab	10.6	-	13.6	2.1	20.8	-
IMbrave150[14,22]	Atezolizumab +Bevacizumab	30*	74*	19.2	6.9	43	2
Finn et al.[8]	Pembrolizumab +Lenvatinib	46*	-	22	8.6	67	3
Leap-002[29]	Pembrolizumab +Lenvatinib	26.1	-	21.2	8.2	62.5	1
Kelley et al.[25]	Tremelimumab +Durvalumab	24	-	18.7	2.2	37.8	-

Checkmate 040[10]	Nivolumab + Ipilimumab	32	-	12.7-22.8	-	-	-
Orient-32[13]	Sintilimab+ bevacizumab biosimilar	21	72	-	4.6	29	0
RESCUE[24]	Camrelizumab +Apatinib	46*<br>25*[#]	76<br>79[#]	20.1<br>21.8[#]	6.4<br>5.5[#]	77.4	1.1

* Evaluated according to mRECIST; [#] Second-line (or more) therapy only.

AE, adverse event; DCR, disease control rate; G≥3, Grade≥3; G5, Grade 5; ICIs: immune checkpoint inhibitors; mPFS, median progression-free survival time; mRECIST, modified response evaluation criteria in solid tumor; MST, median survival time; ORR, objective response rate.
